# Micromechanical modeling of the contact stiffness of an osseointegrated bone–implant interface

**DOI:** 10.1186/s12938-019-0733-3

**Published:** 2019-12-03

**Authors:** Maria Letizia Raffa, Vu-Hieu Nguyen, Guillaume Haiat

**Affiliations:** CNRS, Laboratoire Modélisation et Simulation Multi Echelle, MSME, UMR CNRS 8208, 61 Avenue du Général de Gaulle, 94010 Créteil, France

**Keywords:** Bone–implant interface, Contact, Roughness, Homogenization, Finite element modeling

## Abstract

**Background:**

The surgical success of cementless implants is determined by the evolution of the biomechanical properties of the bone–implant interface (BII). One difficulty to model the biomechanical behavior of the BII comes from the implant surface roughness and from the partial contact between bone tissue and the implant. The determination of the constitutive law of the BII would be of interest in the context of implant finite element (FE) modeling to take into account the imperfect characteristics of the BII. The aim of the present study is to determine an effective contact stiffness $\left( {K_{c}^{\text{FEM}} } \right)$ of an osseointegrated BII accounting for its micromechanical features such as surface roughness, bone–implant contact ratio (BIC) and periprosthetic bone properties. To do so, a 2D FE model of the BII under normal contact conditions was developed and was used to determine the behavior of $K_{c}^{\text{FEM}}$.

**Results:**

The model is validated by comparison with three analytical schemes based on micromechanical homogenization including two Lekesiz’s models (considering interacting and non-interacting micro-cracks) and a Kachanov’s model. $K_{c}^{\text{FEM}}$ is found to be comprised between 10^13^ and 10^15^ N/m^3^ according to the properties of the BII. $K_{c}^{\text{FEM}}$ is shown to increase nonlinearly as a function of the BIC and to decrease as a function of the roughness amplitude for high BIC values (above around 20%). Moreover, $K_{c}^{\text{FEM}}$ decreases as a function of the roughness wavelength and increases linearly as a function of the Young’s modulus of periprosthetic bone tissue.

**Conclusions:**

These results open new paths in implant biomechanical modeling since this model may be used in future macroscopic finite element models modeling the bone–implant system to replace perfectly rigid BII conditions. 
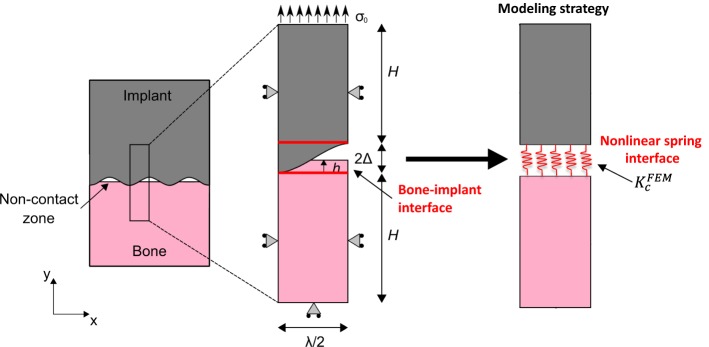

## Background

Endosseous cementless implants have been used clinically for more than 40 years and have allowed considerable progresses in dental, maxillofacial and orthopedic surgery, to replace missing organs or to restore joints functionality. Despite their routine clinical use, implant failures still occur and remain difficult to predict. One of the main determinants of the surgical success lies in the determination of the implant stability [1]. The primary stability occurs during implant surgery. It is a phenomenon of biomechanical nature related to bone quality at the implant site and to the implant properties. A good implant primary stability is a necessary condition to obtain implant osseointegration. Secondary stability is obtained after a certain healing period and corresponds to the initial stability reinforced by newly formed bone production and maturation at the bone–implant interface [2, 3]. Osseointegration phenomena [4] are stimulated by the application of mechanical stimuli to the bone–implant interface (BII). Moreover, the implant surface roughness, which is obtained using different processes such as, for example, sand blasting [5], plasma spraying [6], or laser blasting [7], is known to strongly influence the quality of osseointegration phenomena [8]. When the primary stability is not sufficient, micro-movements may appear, preventing good healing conditions and leading to the formation of fibrous tissue and eventually to surgical failure [9, 10]. Relative micromotions between the implant and bone tissue should not exceed around 150 µm, because it leads to fibrous tissue formation rather than bone ongrowth [11, 12]. Fibrous tissue may develop instead of an osseointegrated interface when there are excessive interfacial micromotions early after surgery [13, 14]. However, micromotions at a relatively low level may be responsible for biomechanical stimulation of bone remodeling.

Consequently, it is important to understand the biomechanical behavior of the BII at the microscopic scale, which depends on the bone geometrical and material properties as well as on the implant surface roughness. Experimental approaches remain of limited interest to retrieve the main determinant of the micromechanics of the BII because many parameters such as the bone–implant contact (BIC) ratio as well as bone material properties are difficult to assess and are likely to vary in parallel. Despite the development of acoustical methods [15, 16] to retrieve information on the BII properties, it remains difficult to employ noninvasive techniques. Classical imaging techniques such as magnetic resonance imaging or X-ray microcomputed tomography cannot be used in vivo due to diffraction effects related to the presence of the titanium [17, 18].

Finite element (FE) models have been widely used to model the implant biomechanical behavior at the organ scale in the context of primary and secondary stability, but often the BII is modeled as a *perfect* interface, i.e., continuity of stresses and displacements at the interface [19]. In particular in the context of dental implantology, microfinite element analyses were applied to images obtained using X-ray micro-computed tomography [20, 21], which allowed to assess the strain and stress field around the implant. However, the BII was often considered as fully bonded. Some groups modeled the BII during osseointegration as an interphase, considering a thin layer described by the Drucker–Prager plasticity model [22]. The use of springs to model the BII was introduced by Egan and Marsden [23], who described load transfer at the BII by a network of linear springs whose stiffness can vary in time.

However, it remains difficult to relate the macroscopic behavior of the BII in terms of contact stiffness [23] or continuous mechanical properties [22] to its micromechanical properties such as the BIC and bone properties. Establishing such a relationship would be interesting in the context of large-scale finite element (FE) simulations because it could allow to replace the BII by an interface constitutive law (soft imperfect interface [24]) instead of considering a rigid interface behavior (perfect interface). Such an approach will allow to scale the BII microscopic properties up to the macroscopic behavior in a multiscale FE-simulation framework.

The aim of this study is to develop a multiscale model of the biomechanical behavior of an osseointegrated BII, which is modeled as an imperfect interface, taking into account its microscopic properties: the BIC ratio, the implant surface roughness and the bone properties. An effective incremental contact stiffness in normal direction is numerically derived and compared to analytical predictions.

## Results

In “Voigt–Reuss bounds” section, the numerical contact stiffness $K_{c}^{\text{FEM}}$ is compared with the analytical stiffness obtained from the Voigt–Reuss bounds. In “Comparisons with the analytical models” section, the results obtained with the FEM (described in “Finite element modeling” section) and the three analytical models (described in “Analytical approaches” section) are compared. Finally, the parametrical analyses investigating the effect of the BIC, of the roughness amplitude ∆, of the roughness wavelength *λ* and of the bone Young’s modulus *E*_*b*_ are presented in “Parametrical analyses” section.

### Voigt–Reuss bounds

**Fig. 1:**
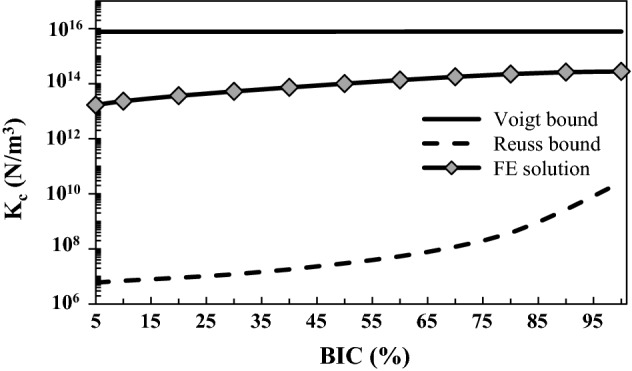
Comparison between the numerical effective contact stiffness $K_{c}^{\text{FEM}}$ and the analytical stiffness obtained from the Voigt–Reuss bounds [see Eqs. (7)**–**(10)] as a function of the BIC. The roughness wavelength *λ*, amplitude ∆ and the bone Young’s modulus *E*_*b*_ are equal to their reference values 80 µm, 5 µm and 2 GPa, respectively

Figure 1 shows the variation of the effective contact stiffness obtained using the numerical approach described in “Numerical resolution” section as a function of the BIC in the reference configuration (∆ = 5 µm, *λ* = 80 µm, *E*_*b*_ = 2 GPa), which is compared with the Voigt–Reuss bounds. As expected, the numerical results are comprised between Voigt (upper) and Reuss (lower) bounds for different BIC scenarios, which constitutes a first validation of the numerical model.

### Comparisons with the analytical models

**Fig. 2:**
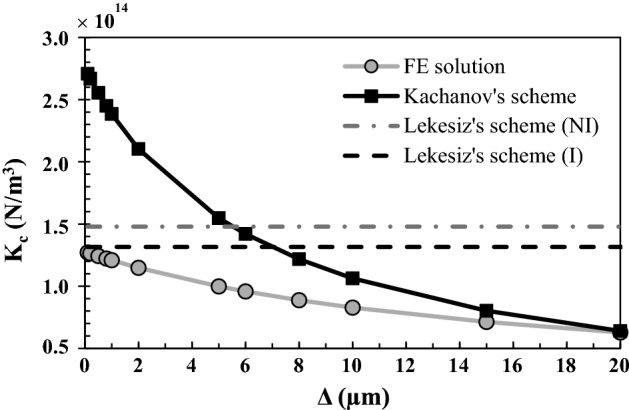
Variation of the effective contact stiffness $K_{c}$ obtained with the finite element model, the Kachanov’s model, the Lekesiz’s model with crack interaction (I) and the Lekesiz’s model without crack interaction (NI) as a function of the roughness amplitude ∆. The roughness wavelength *λ*, the BIC and the bone Young’s modulus *E*_*b*_ are equal to their reference values 80 µm, 50% and 2 GPa, respectively

Figure 2 shows the variation of the effective contact stiffness $K_{c}$ obtained with the finite element model (gray solid line), the Kachanov’s model (black solid line), the Lekesiz’s model with crack interaction (black dashed line) and the Lekesiz’s model without crack interaction (gray dashed line) as a function of the roughness amplitude ∆. All other parameters are taken equal to their reference value. The effective contact stiffness is shown to decrease as a function of ∆ for the FEM and for the Kachanov’s model. Moreover, as expected, the results obtained with both Lekesiz’s models do not depend on ∆.

**Fig. 3:**
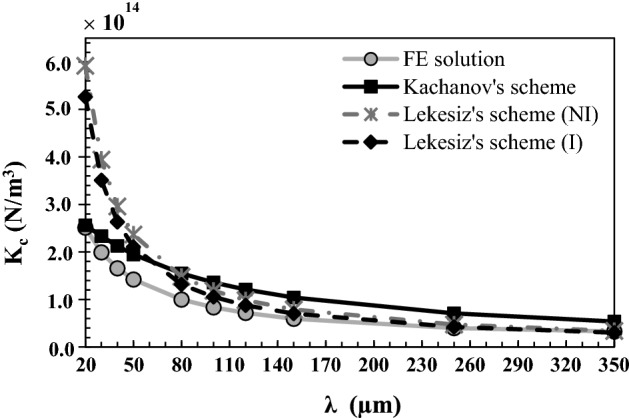
Variation of the effective contact stiffness $K_{c}$ obtained with the finite element model, the Kachanov’s model, the Lekesiz’s model with crack interaction (I) and the Lekesiz’s model without crack interaction (NI) as a function of the roughness wavelength *λ*. The roughness amplitude ∆, the BIC and the bone Young’s modulus *E*_*b*_ are equal to their reference values 5 µm, 50% and 2 GPa, respectively

Figure 3 shows the variation of the effective contact stiffness $K_{c}$ obtained with the finite element model (gray solid line), the Kachanov’s model (black solid line), the Lekesiz’s model with crack interaction (black dashed line) and the Lekesiz’s model without crack interaction (gray dashed line) as a function of the roughness wavelength *λ*. All other parameters are taken equal to their reference values. The effective contact stiffness is shown to decrease as a function of *λ* for all four models.

**Fig. 4:**
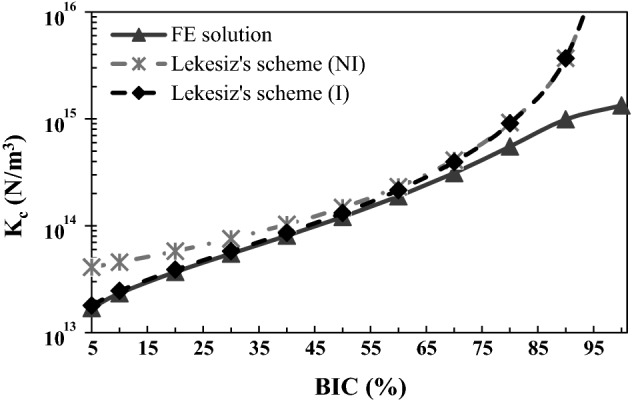
Variation of the effective contact stiffness $K_{c}$ obtained with the finite element model, the Lekesiz’s model with crack interaction (I) and the Lekesiz’s model without crack interaction (NI) as a function of the BIC. The roughness amplitude ∆ is equal to 1 µm; the roughness wavelength *λ* and the bone Young’s modulus *E*_*b*_ are equal to their reference values 80 µm and 2 GPa, respectively

Figure 4 shows the variation of the effective contact stiffness $K_{c}$ obtained with the finite element model (gray solid line), the Lekesiz’s model with crack interaction (black dashed line) and the Lekesiz’s model without crack interaction (gray dashed line) as a function of the BIC. In this case, the numerical stiffness has been calculated assuming ∆ = 1 µm to compare the FE solution with the analytical solutions by Lekesiz, which are valid in the case of crack-like non-contact zones (see “Lekesiz’s models” section). All other parameters in the FE model are taken equal to their reference values. The effective contact stiffness is shown to increase as a function of the BIC for all three models.

### Parametrical analyses

**Fig. 5:**
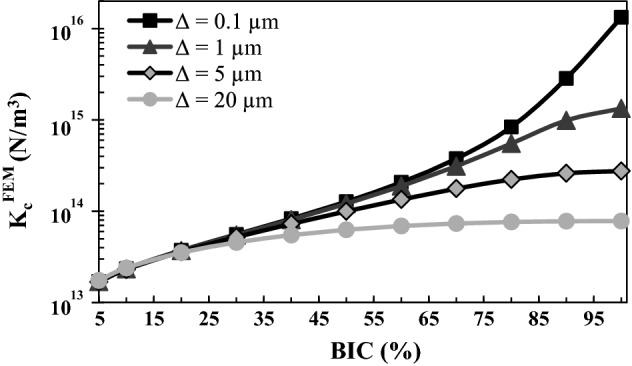
Variation of the numerical effective contact stiffness $K_{c}^{\text{FEM}}$ as a function of the BIC for different values of the roughness amplitude ∆. The roughness wavelength *λ* and the bone Young’s modulus *E*_*b*_ are equal to their reference values 80 µm and 2 GPa, respectively

Figure 5 shows the variation of the numerical effective contact stiffness $K_{c}^{\text{FEM}}$ as a function of the BIC for different values of ∆. The wavelength *λ* and the bone Young’s modulus *E*_*b*_ are equal to their reference values 80 µm and 2 GPa, respectively. $K_{c}^{\text{FEM}}$ is shown to increase as a function of the BIC for all values of ∆ considered. The sensitivity of $K_{c}^{\text{FEM}}$ to BIC variations is more important for lower values of ∆ and high values of the BIC. For low BIC values, $K_{c}^{\text{FEM}}$ weakly depends on ∆; while for high BIC values, $K_{c}^{\text{FEM}}$ decreases significantly as a function of ∆.

**Fig. 6:**
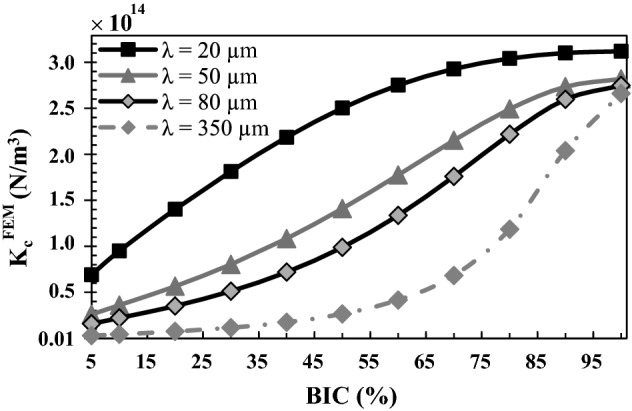
Variation of the numerical effective contact stiffness $K_{c}^{\text{FEM}}$ as a function of the BIC for different values of *λ*. The roughness amplitude ∆ and the bone Young’s modulus *E*_*b*_ are equal to their reference values 5 µm and 2 GPa, respectively

Figure 6 shows the variation of the numerical effective contact stiffness $K_{c}^{\text{FEM}}$ as a function of the BIC for different values of *λ*. The roughness amplitude ∆ and the bone Young’s modulus *E*_*b*_ are equal to their reference values 5 µm and 2 GPa, respectively. $K_{c}^{\text{FEM}}$ is shown to increase as a function of the BIC for all values of *λ* considered. Moreover, $K_{c}^{\text{FEM}}$ decreases significantly as a function of *λ* for all BIC values.

**Fig. 7:**
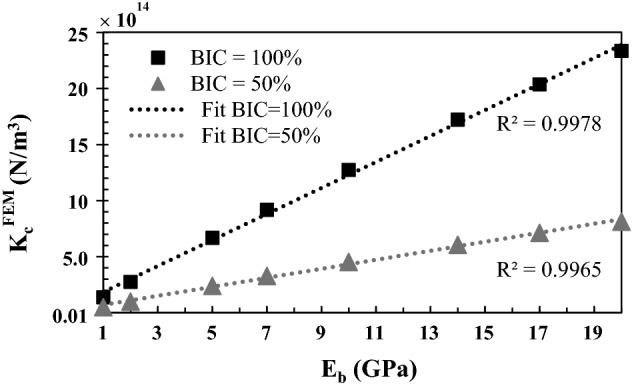
Variation of the numerical effective contact stiffness $K_{c}^{\text{FEM}}$ as a function of the bone Young’s modulus *E*_*b*_ for different values of the BIC. The roughness amplitude ∆ and the wavelength *λ* are equal to their reference values 5 µm and 80 GPa, respectively

Figure 7 shows the variation of the numerical effective contact stiffness $K_{c}^{\text{FEM}}$ as a function of the bone Young’s modulus *E*_*b*_ for BIC = 50% and BIC = 100%. The roughness amplitude ∆ and the roughness wavelength *λ* are equal to their reference values 5 µm and 80 µm, respectively. $K_{c}^{\text{FEM}}$ is shown to increase linearly as a function of *E*_*b*_ for all BIC values considered. The slope of the variation of $K_{c}^{\text{FEM}}$ as a function of *E*_*b*_ is higher for BIC = 100% compared to the case where BIC = 50%.

## Discussion

The originality of this work is to provide a homogenized interface model able to obtain an effective normal contact stiffness of the BII accounting for bone ingrowth and implant micro-roughness. The proposed 2D numerical model was validated by comparison with five analytical models. The Lekesiz’s schemes were employed to describe the asymptotic limit behavior of the proposed FE-based interface model when the aspect ratio of the non-contact zone ${b \mathord{\left/ {\vphantom {b a}} \right. \kern-0pt} a}$ tends to zero, which corresponds to the case where the non-contact zones can be modeled as internal micro-cracks. The results obtained with Lekesiz’s models were also employed to highlight the effect of the interaction between non-contact zones. Kachanov’s scheme was used to describe the asymptotic limit behavior when the aspect ratio of the non-contact zone ${b \mathord{\left/ {\vphantom {b a}} \right. \kern-0pt} a}$ tends to 1 which corresponds to the case where the non-contact zones can be modeled as elliptical holes. The results presented herein show that the roughness parameters (∆ and *λ*), the bone Young’s modulus Eb as well as the BIC have a significant effect on the effective contact stiffness of the BII.

Figure 7 shows that the effective normal contact stiffness increases linearly as a function of the bone Young’s modulus, which may be explained by considering the Hertzian contact problem between two elastic spheres made of the same isotropic material (*E*, $\nu$) [25]. In the Hertzian theory, for a circular contact area *S*, the incremental normal contact stiffness $K_{N}^{c}$ per contact area, reads as follows [26]:

1 $$K_{N}^{c} = \frac{E}{{1 - \nu^{2} }}\sqrt {\frac{S}{\pi }} .$$

Equation (1) shows that the contact stiffness $K_{N}^{c}$ is proportional to the material Young’s modulus *E*, which explains the linearity of $K_{c}^{\text{FEM}}$ as a function of the bone Young’s modulus *E*_*b*_ obtained in Fig. 7. Moreover, Eq. (1) shows that the normal contact stiffness increases as a function of *S*, which is directly related to the BIC, thus explaining that the slope of the variation of $K_{c}^{\text{FEM}}$ as a function of *E*_*b*_ is higher for BIC = 100% compared to the case where BIC = 50%.

Voigt–Reuss bounds are obtained by a rule of mixtures as a weighted average, and they provide the theoretical widest upper- and lower bounds used to predict various properties (mechanical, thermal, etc.) of fiber-composite materials and porous materials. The effective elastic properties obtained by a homogenization technique must fall into these bounds. Accordingly, results shown in Fig. 1 constitute a first validation for the proposed homogenized numerical model. In addition, the analytical stiffness obtained by both Lekesiz’s and Kachanov’s schemes are comprised within Voigt–Reuss bounds as can be highlighted by comparing Figs. 1, 2, 3 and 4.

**Fig. 8:**
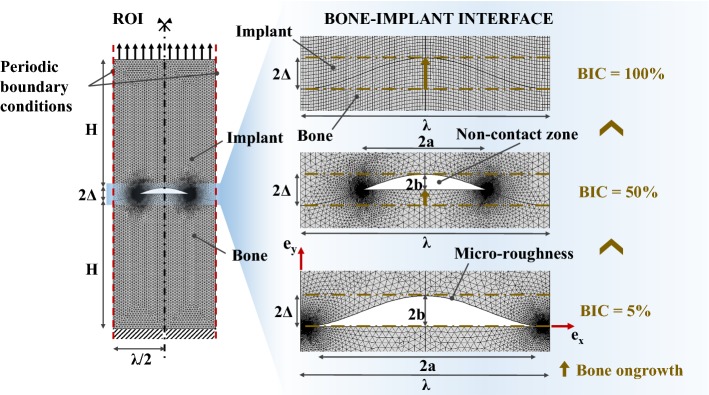
Mechanical and geometrical configurations of the FE model. Three phases of the bone ongrowth are sketched, corresponding to BIC = 5%, 50% and 100%

The results shown in Figs. 1, 4, 5 and 6 indicate that the normal contact stiffness obtained by FE analyses $K_{c}^{\text{FEM}}$ always increases as a function of the BIC. Note that a similar behavior is obtained in the Reuss model (see Fig. 1), as well as with the Lekesiz’s models (see Fig. 4). These results may be explained by the fact that increasing of the BIC leads to a more important contact area, which is known to increase contact rigidity nonlinearly (refer to the Hertzian contact model Eq. (1), for instance). Moreover, increasing the BIC leads to a decrease of the void fraction (see Fig. 8), which also leads to a more rigid interface behavior.

The results shown in Figs. 2 and 5 indicate that the contact stiffness $K_{c}^{\text{FEM}}$ decreases as a function of ∆, for BIC > 20%. This result may be explained by the increase of the size of the cavity in the *y*-direction when ∆ increases, leading to lower rigidity of the system compared to a cavity with a lower aspect ratio (defined by ${b \mathord{\left/ {\vphantom {b a}} \right. \kern-0pt} a}$). Note that similar results were obtained with the Kachanov’s model (see Fig. 2), which may be explained by the same analysis. Other authors have also found a higher normal contact stiffness for lower values of the roughness amplitude with analytical models [27] and experimental approaches [28].

For BIC values lower than around 20%, the contact stiffness $K_{c}^{\text{FEM}}$ weakly depends on the amplitude ∆, as shown in Fig. 5. In this case, the overall contact is concentrated near the peaks of the implant surface (*y* = 0 and *y* = *λ*/2 in Fig. 8), which present an initially horizontal slope due to the sinusoidal geometry. Therefore, for low values of BIC, such situation may be approximated by a “flat punch” configuration, which does not depend on variations of ∆ because the effective contact area does not vary significantly.

When the BIC is equal to 100%, the porosity of the BII vanishes (see Fig. 8); so, the numerical interface model acts like a bi-material layer. In this case, as shown in Fig. 5, the overall stiffness is shown to increase when ∆ decreases, as expected for a thin elastic layer [24].

The results shown in Fig. 6 indicate that the contact stiffness $K_{c}^{\text{FEM}}$ decreases as a function of *λ*, which may be explained by the decrease of the contact area (or contact length in our 2D model) per unit length (along the *x*-direction) when the wavelength *λ* increases. This behavior can be analytically explained by the expression of the contact length per unit length $\frac{{L_{P} }}{\lambda } = \frac{1}{\pi }E\left( { - \frac{{4\pi^{2} \Delta^{2} }}{{\lambda^{2} }}} \right)$, obtained by Eq. (4) after some algebra.

The numerical and analytical results may be compared in specific configurations. First, the cases where (i) ∆ tends towards 0 and (ii) *λ* increases significantly both correspond to an aspect ratio of the non-contact zone ${b \mathord{\left/ {\vphantom {b a}} \right. \kern-0pt} a}$ tending towards 0. Such situation corresponds in turn to the micro-crack of the Lekesiz’s model, which explains why the effective contact stiffness obtained with the FEM tends towards that obtained with the Lekesiz’s model with crack interaction when ∆ tends towards 0 (see Fig. 2) as well as when *λ* > 200 µm (see Fig. 3). Second, the cases where (i) ∆ increases significantly and (ii) *λ* tends towards 0 correspond to aspect ratio of the non-contact zone ${b \mathord{\left/ {\vphantom {b a}} \right. \kern-0pt} a}$ tending towards 1. This situation corresponds in turn to Kachanov model, which explains why the effective contact stiffness obtained with the FEM tends towards that obtained with the Kachanov model for *λ* < 30 µm (see Fig. 3) as well as when ∆ > 15 µm (see Fig. 2).

Figure 4 shows that for relatively low BIC values (below around 50%), the results obtained with the Lekesiz’s model with crack interaction (I) and with the FE model are in good agreement, while the results obtained with the Lekesiz’s model without crack interaction (NI) overestimates the contact stiffness obtained with the FE model. When considering high BIC values within the physiological range (50% < BIC < 75%), the results obtained with the Lekesiz’s model without crack interaction (NI) and with the FE model are in good agreement. This result may be explained by the fact that the distance between two contiguous non-contact zones (i.e., $\lambda - 2a$) increases as a function of the BIC (see Fig. 8). Note that for BIC > 50% the analytical solutions obtained by models with (I) and without (NI) interactions overlap because the interaction parameter $I_{k} \left( {\frac{a}{\lambda }} \right)$ expressed by Eq. (16) is equal to 1. Physically, this finding is in agreement with the theory of the micro-cracked media, which establishes that the interaction between cracks could be neglected when the distance between two contiguous cracks is greater or equal to their length [52]. Therefore, when BIC values are relatively low, a suitable choice is to consider interacting cracks, while cracks interactions may be neglected for relatively high BIC values. Note that when the BIC tends towards 100% (which corresponds to $a$ tending to 0), both Lekesiz’s models becomes invalid giving an infinite contact stiffness because of the interaction parameter $I_{k} \left( {\frac{a}{\lambda }} \right)$.

Several parameters were chosen empirically. First, the choice of the thickness *H* = 500 µm of the layers representing bone tissue and the implant was made to find a compromise between a sufficiently low value to obtain reasonable computational time and a sufficiently high value so that the results do not depend on *H* for all configurations. Accordingly, a parametric study on *H* was carried out in the most unfavorable case that is for *λ* = 350 µm. The numerical contact stiffness was found not to depend on *H* for *H* > 300 µm.

Second, all numerical simulations have been carried out considering a uniform normal tensile stress $\sigma_{0}$ = 25 MPa (see Fig. 8), which was chosen because such amount of stress has been shown to be obtained in clinical configuration [29]. However, it has been verified that the results do not depend on the choice of the value of $\sigma_{0}$, which is explained by the linear assumptions of the model.

This study has several limitations. First, the FE model was developed in 2D and the BII description should be realized considering the three dimensions, which could be done using approaches such as, for example, 3D analytical approaches based on the Eshelby theory [30–32]. Second, a sinusoidal function is used to describe the implant surface roughness, similarly as what was done by [46]. This sinusoidal description of the implant surface constitutes a strong approximation and considering the real surface texture is likely to lead to different results. Note that comparable approaches using wavy surfaces have already been developed in contact mechanics in the past [33]. Third, only normal stresses were considered and assessing the influence of shear stresses would be of interest since it corresponds to a situation of clinical interest. Fourth, bone material properties were assumed to be linearly elastic, homogeneous and isotropic, and fluid structure interactions were neglected, similarly as what was done in various previous FE-based numerical studies [34–38]. However, newly formed bone properties are heterogeneous [39] and viscoelastic, which was neglected herein. The assumption of bone homogeneity implies a simplified modeling of the bone microstructure, which may include cavities at various scales [40, 41]. Note that adopted analytical approaches can be extended to anisotropy of the bone by considering microcracks and pores embedded into an anisotropic matrix. Moreover, a dry-material model was employed and fluid–structure interaction effects were neglected; these assumptions were necessary to understand the main contact mechanisms occurring at the BII, by simplifying as coherently as possible the biomechanical problem. Note that in vivo, fully bonded interfaces are not likely to occur since values of the BIC are typically comprised between around 30 and 80% [42–44]. Fifth, the proposed model should be validated experimentally. However, accurate data on bone–implant contact stiffness are scarce because of the difficulty of controlling the implant surface as well as the bone distribution around the implant. However, a validation of the proposed numerical model was carried out by comparison with three analytical models (and Voigt–Reuss bounds). Sixth, adhesion phenomena at the BII [45] that may be important in particular at early stage of osseointegration were not taken into account, which should be considered in the future.

Despite the aforementioned limitations, this work constitutes the first attempt to determine an effective normal contact stiffness for an osseointegrated BII, which is able to account for the microstructural effects of periprosthetic bone properties and implant micro-roughness.

## Conclusions

The present study proposes a nonlinear spring model for an osseointegrated bone–implant interface in normal contact conditions based on micromechanical modeling. A 2D FE model accounting for implant micro-roughness and bone ongrowth is used to obtain an effective incremental contact stiffness $K_{c}^{\text{FEM}}$ in normal direction. Several parametrical analyses have been carried out to investigate the effects of the micro-roughness parameters Δ and $\lambda$, the BIC ratio and the bone Young’s modulus *E*_*b*_, on $K_{c}^{\text{FEM}}$. Comparisons with analytical schemes based on micromechanical homogenization (Eshelby’s problem [58]) have been employed to validate the FE model, as well as to provide an insight on the advantages and limitations of closed-form analytical solutions for the estimation of the effective contact stiffness of BII. The proposed nonlinear spring modeling strategy for BII can allow to overcome computational difficulties to account for BII evolving microstructure within large-scale finite element (FE) simulations.

Future work should focus on 3D modeling (considering actual surface profiles), on the tangential movement and on studying the biomechanical reaction terms which mimic osseointegration phenomena.

## Methods and models

Let the general framework of the contact problem be introduced by considering two continuous bodies (representing in the following implant and bone tissue) comprising linearly elastic isotropic materials, in *no*-*sliding contact* via a rough surface under a tensile loading condition (Fig. 8). Let a Cartesian frame (*O*, **e**_x_, **e**_y_, **e**_z_) be introduced, with *x*, *y* and *z* the corresponding coordinates. Let $S \subset {\mathbb{R}}^{2}$ be the nominal contact area between the two bodies. Then, the normal incremental contact stiffness per unit nominal contact area in $S$ is defined as:

2 $$K_{N}^{c} = \frac{{dF_{N} }}{dw},$$

where $dw$ is the increment of the relative displacement at the contacting interface region in normal (i.e., along **e**_y_) direction, and $dF_{N}$ is the increment of the normal force transmitted through the unit contact area.

### Finite element modeling

#### Bone–implant interface modeling

The contact region of interest (ROI) comprises two subdomains corresponding to the bone tissue and to the implant with the same thickness *H* = 500 µm (along **e**_y_). Similarly as what was done by [46], a simplistic idealization of the contacting rough implant surface via a sinusoidal wavy-like surface, of amplitude 2Δ and wavelength *λ*, is adopted:

3 $$y\left( x \right) = \Delta \left[ {1 - \cos \left( {\frac{2\pi x}{\lambda }} \right)} \right],\quad x \in \left[ {0, \lambda } \right].$$

Since the out-of-plane dimension of the ROI (i.e., along **e**_z_) is assumed to be infinite and applied load is homogeneous, an assumption of plane strain is considered herein, in the plane (**e**_x_, **e**_y_).

The BIC ratio (i.e., the ratio of the bone in direct contact with the implant surface) which is assumed to be comprised between 5 and 100% depends on the bone ongrowth onto the implant surface and is an input parameter of the model. Non-contact zones between bone and the implant determine the microstructural voids of the BII. The dimension of these voids along **e**_y_ (respectively, along **e**_x_) is noted *2b* (respectively, *2a*) and depends on $\Delta ,\lambda$ and BIC. Note that for the proposed model, the BIC is geometrically given by:

4 $${\text{BIC}} = \frac{{L_{P} }}{{L_{T} }} = 1 - \frac{{E\left( {\left. {\frac{2\pi a}{\lambda }} \right| - \frac{{4\pi^{2} \Delta^{2} }}{{\lambda^{2} }}} \right)}}{{2E\left( { - \frac{{4\pi^{2} \Delta^{2} }}{{\lambda^{2} }}} \right)}},$$

where *L*_*P*_ (respectively, *L*_*T*_) is the arc length of the implant surface in contact with bone tissue (respectively, the total arc length of the implant boundary), and $E\left( z \right) = E\left( {\frac{\pi }{2}|z} \right)$ and $E = \left( {z|m} \right) = \int_{0}^{z} {\sqrt {1 - m\sin^{2} \left( t \right)} {\text{d}}t}$ represent the complete and incomplete elliptic integral of the second kind [47], respectively.

The material properties of the implant and bone tissue are assumed to be linear elastic, isotropic and homogeneous. The implant is assumed to be made of titanium alloy (TiAl6V4) with a Young’s modulus *E*_*i*_ = 113 GPa [48]. The bone Young’s modulus *E*_*b*_ is varied between 1 and 20 GPa, to simulate the increase of the stiffness of the healing periprosthetic bone tissue due to osseointegration phenomena [49]. All materials are assumed to have a Poisson ratio *ν* of 0.3.

Figure 8 shows the boundaries conditions for the proposed FE model. A symmetric boundary condition ${\mathbf{u}} \cdot {\mathbf{e}}_{{\mathbf{x}}} = u_{x} = 0$ holds on the boundaries parallel to **e**_y_, where ${\mathbf{u}}$ is the displacement vector. The lower boundary (*y *= **− ***H*) of the bone domain is fixed $({\mathbf{u}} = 0)$. A uniform normal tension $\sigma_{0}$ = 25 MPa is applied on the upper boundary of the implant domain (*y *= 2∆ + *H*) for all FE analyses. The choice of these model parameters is discussed in “Discussion” section. Continuity conditions of the displacement and of the traction (bounded interface condition) hold at the contacting surfaces between bone and implant.

#### Numerical resolution

The goal of the proposed FE simulations is to derive the incremental contact stiffness in the normal direction of the BII, denoted $K_{c}^{\text{FEM}}$. To do so, a homogenization model of the interphase representing the bone–implant contacting zone (see Fig. 8) was developed to derive the normal incremental contact stiffness. An equivalent one-dimensional system comprising three springs in series representing the implant domain (spring stiffness *K*_*i*_), the bone domain (spring stiffness *K*_*b*_) and the BII (spring stiffness $K_{c}^{\text{FEM}}$) was considered. The stiffness of the one-dimensional bone–implant system *K*_eq_ described above is determined numerically following:

5 $$K_{\text{eq}} = \frac{{\left\langle {\sigma_{yy} } \right\rangle }}{{\left\langle {{\text{u}}_{y} } \right\rangle }},$$

where $\sigma_{yy} = \left( {{\varvec{\upsigma}} {\mathbf{e}}_{{\mathbf{y}}} } \right) \cdot {\mathbf{e}}_{{\mathbf{y}}}$ and ${\text{u}}_{y}$ are, respectively, the stress and displacement along **e**_y_ and $\left\langle \bullet \right\rangle = \frac{2}{\lambda }\int { \bullet \,{\text{d}}x}$ represents the average on the loaded boundary (at *y *= $2\Delta$ + *H*, see Fig. 1). As a result, the numerical contact stiffness of the BII is given by:

6 $$K_{c}^{\text{FEM}} = \left[ {K_{\text{eq}}^{ - 1} - \left( {K_{i}^{ - 1} + K_{b}^{ - 1} } \right)} \right]^{ - 1} ,$$

where $K_{j} = \frac{{\lambda_{j} + 2\mu_{j} }}{\delta }; \; j = i,b$ (the subscript *i* and *b* denotes the implant and bone tissue, respectively). In Eq. (6), $\lambda_{j}$ and $\mu_{j}$ are the Lamé parameters of the implant and bone materials and $\delta = H - \Delta$ corresponds to the size of the implant and bone domain.

Equation 6 is used to obtain the numerical contact stiffness of the BII for each FE-based simulation. Several parametrical analyses have been carried out to investigate the influence of the BIC (between 5 and 100%), of the roughness amplitude ∆ (between 0.1 and 20 µm), of the roughness wavelength *λ* (between 20 and 350 µm) and of the bone Young’s modulus *E*_*b*_ (between 1 and 20 GPa). The ranges of variation of ∆ and *λ* were chosen based on the values of the arithmetic mean roughness (*R*_*a*_) and the mean spacing (*S*_*m*_) measured on real titanium implants [10], noting that ${\text{R}}_{\text{a}} \cong 2\Delta /\pi$. The reference configuration corresponds to the following parameters: BIC = 50%, ∆ = 5 µm, *λ* = 80 µm and *E*_*b*_ = 2 GPa.

The numerical analyses have been carried out using the Comsol Multiphysics^®^ simulation software (Stockholm, Sweden). The total mesh comprises approximately 9300 second-order triangular Lagrange elements depending on the geometrical configuration of the micro-roughness parameters ∆ and *λ* (e.g., 9274 in the reference configuration). The global system comprises about 38,000° of freedom (e.g., 37,742 in the reference configuration). For the sake of regularity, a mapped second-order quadrangular mesh has been chosen only for the case BIC = 100%, as shown in Fig. 8. The interpolation functions for the displacement field are quadratic. The mesh size has to be finer around the tip of the non-contact zone, as shown in Fig. 8, to describe the stress localization, especially for a smaller interface thickness 2∆. To this aim, a convergence study was performed to choose the mesh size at the tip in the most unfavorable configuration that is for ∆ = 0.1 µm. The chosen mesh has a smallest element size of 3 × 10^−5^ µm. The local error on the approximated solution ${\text{u}}_{y}^{a}$ calculated in terms of the displacement ${\text{u}}_{y}$ is $\frac{{\left\| {{\text{u}}_{y}^{e} - {\text{u}}_{y}^{a} } \right\|_{{L^{2} }} }}{{\left\| {{\text{u}}_{y}^{e} } \right\|_{{L^{2} }} }} = 0. 0 0 6 {\text{\%,}}$ where $\left\| \bullet \right\|_{{L^{2} }}$ indicates the *L*^2^ norm.

### Analytical approaches

**Fig. 9:**
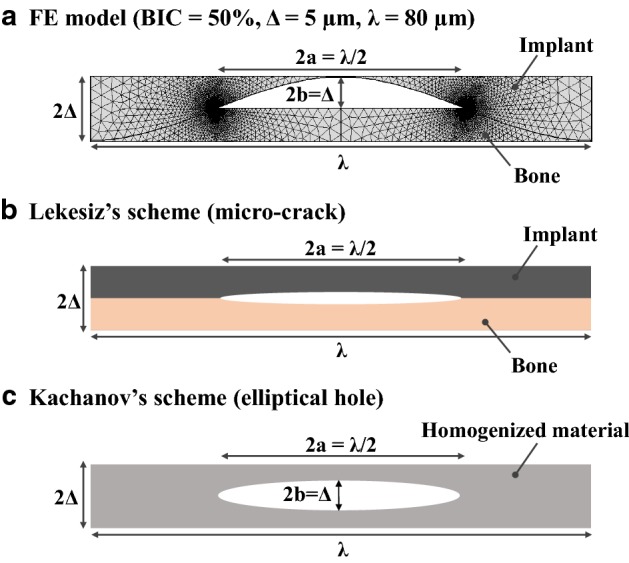
Schematic representation of the BII for **a** the numerical and **b**, **c** the analytical models. FEM-based interface model in the reference configuration **a** is compared with two analytical descriptions: **b** the Lekesiz’s scheme and **c** the Kachanov’s scheme. Dimensions 2*a* and 2*b* are specified in the three models for BIC = 50%

The non-contact zone of the BII (Fig. 9a) may be approximated by an *internal* micro-crack [50] when ${b \mathord{\left/ {\vphantom {b a}} \right. \kern-0pt} a} \to 0$, as shown in Fig. 2b. Lekesiz’s schemes consider an array of planar cracks at the interface between two dissimilar isotropic materials [51] under two hypotheses assuming i) interacting and ii) non-interacting micro-cracks.

Conversely, the non-contact zone may be better approximated by an elliptical cavity [52] when its aspect ratio ${b \mathord{\left/ {\vphantom {b a}} \right. \kern-0pt} a} \to 1$, as shown in Fig. 2c. Kachanov’s scheme considers elliptical holes randomly oriented embedded into an isotropic homogenized material [53] in the non-interacting approximation [54].

#### Voigt and Reuss bounds

The Voigt and Reuss bounds represent the upper and the lower limits for the homogenized mechanical properties of a multi-phase composite material, respectively. These analytical bounds have been calculated considering the region of interest shown in Fig. 9a and constituted by the implant, bone tissue and the void corresponding to the non-contact zone (for BIC < 100%). For BIC = 100%, a two-phase medium made of bone and implant was considered. The area fractions of bone tissue, implant and void were noted $f_{b}$, $f_{i}$ and $f_{\text{void}}$, respectively, and were related to the BIC, ∆ and *λ*. The analytical contact stiffness given by the Voigt bound writes [24]:

7 $$K_{c}^{V} = \frac{1}{2\Delta }\left( {\kappa_{V} + \frac{4}{3}\mu_{V} } \right) ,$$

where $\kappa_{V}$ and $\mu_{V}$ are the equivalent bulk and shear moduli, respectively, given as a function of the bulk moduli and shear moduli of bone tissue $\kappa_{b} , \mu_{b}$; implant $\kappa_{i} , \mu_{i}$ and void $\kappa_{\text{void}} , \mu_{\text{void}}$, as follows:

8 $$\begin{aligned} & \kappa_{V} = f_{i} \kappa_{i} + f_{b} \kappa_{b} + f_{\text{void}} \, \kappa_{\text{void}} , \\ & \mu_{V} = f_{i} \mu_{i} + f_{b} \mu_{b} + f_{\text{void}} \, \mu_{\text{void}} , \\ \end{aligned}$$

Similarly, the analytical contact stiffness given by the Reuss bound is given by:

9 $$K_{c}^{R} = \frac{1}{2\Delta }\left( {\kappa_{R} + \frac{4}{3}\mu_{R} } \right) ,$$

where $\kappa_{R}$ and $\mu_{R}$ are the equivalent bulk modulus and shear modulus, respectively, given by:

10 $$\begin{aligned} & \kappa_{R} = \left( {f_{i} \kappa_{i}^{ - 1} + f_{b} \kappa_{b}^{ - 1} + f_{\text{void }} \kappa_{\text{void}}^{ - 1} } \right)^{ - 1} , \\ & \mu_{R} = \left( {f_{i} \mu_{i}^{ - 1} + f_{b} \mu_{b}^{ - 1} + f_{\text{void }} \mu_{\text{void}}^{ - 1} } \right)^{ - 1} . \\ \end{aligned}$$

In Eqs. (8) and (10), the bulk modulus and shear modulus of the void are assumed to be equal to those of a very soft material of which the elastic moduli are taken as $\kappa_{\text{void}} = \varphi \, \kappa_{b}$ and $\mu_{\text{void}} = \varphi \,\mu_{b}$, with $\varphi$ = 10^−9^, following what has been done in [55]. Note that hanging the value of $\varphi$ between 10^−12^ and 10^−6^ does not affect the results.

#### Lekesiz’s models

Lekesiz’s scheme [51] considers two different media separated by a partial non-contact zone modeled as an internal micro-crack of length 2*a.* Note that 2*a* = *λ*/2 for BIC = 50% (see Fig. 2b). Lekesiz provided a closed-form analytical expression for the effective spring stiffness of a planar periodic array of collinear cracks at the interface between two dissimilar isotropic materials. Lekesiz’s scheme is based on an elastic analysis under normal loading in the framework of the open crack model [56]. Lekesiz also provided an analytical expression of the effective contact stiffness taking into account the effects of the interaction between cracks. Two models taken from [51] were considered in this study.

*Interacting micro*-*cracks* When the distance between non-contact zones is small compared to *λ*, these non-contact zones can be assumed to interact with each other. In this case, the effective contact stiffness $K_{{c, {\text{int}}}}^{\text{LEK}}$ is:

11 $$K_{{c, \text{int} }}^{\text{LEK}} = M_{k} \left( {\alpha , \beta } \right) \times I_{k} \times K_{{c , {\text{non-int}}}}^{\text{homog}} ,$$

where the elastic dissimilarity function $M_{k} \left( {\alpha , \beta } \right)$ is expressed in terms of the Dundurs’ parameters $\alpha$ and $\beta$ within the assumption of plane strain [57] following:

12 $$M_{k} \left( {\alpha ,\beta } \right) = \frac{{\left( {1 + \alpha } \right)}}{{\left( {1 - \beta^{2} } \right)\left( {1 + 4 \in^{2} } \right)}},$$

13 $$\alpha = \frac{{E_{b}^{*} - E_{i}^{*} }}{{E_{b}^{*} + E_{i}^{*} }},$$

14 $$\beta = \frac{1}{2}\left( {\frac{{\mu_{b} - \mu_{i} }}{{\mu_{b} + \mu_{i} }}} \right)\left( {\frac{1 - 2\nu }{1 - \nu }} \right),$$

15 $$\in = \frac{1}{2\pi }\log \left( {\frac{1 + \beta }{1 - \beta }} \right),$$

where $\in$ is the oscillation index and $E_{j}^{*} = \frac{{E_{j} }}{{1 - \nu^{2} }}; \quad j = i,b$. The interaction parameter $I_{k} \left( {\frac{a}{\lambda }, \in } \right)$ can be approximated by the homogeneous case $I_{k} \left( {\frac{a}{\lambda }} \right)$ when $\left| \in \right| < 0.05$. In the proposed model, the oscillation index is equal to $\left| \in \right| = 0.039$. Therefore, the interaction parameter is:

16 $$I_{k} \left( {\frac{a}{\lambda }} \right) = \frac{{\pi^{2} }}{8}\left( {\frac{a}{\lambda }} \right)^{2} \left\{ {\ln \left[ {\sec \left( {\frac{\pi a}{2\lambda }} \right)} \right]} \right\}^{ - 1} ,$$

where $\frac{a}{\lambda }$ represents the linear crack density, as sketched in Fig. 9.

The parameter $K_{{c ,\,{\text{non-int}}}}^{\text{homog}}$ in Eq. (11) corresponds to the contact stiffness in the case of non-interacting cracks embedded in a homogeneous domain, assumed to be the implant material, and reads:

17 $$K_{{c ,\,{\text{non-int}}}}^{\text{homog}} = \frac{8}{\pi }\frac{\lambda }{{a^{2} }}\frac{{\mu_{i} }}{{\left( {1 + \gamma_{i} } \right)}},$$

where $\gamma_{i} = 3 - 4\nu$ is the Kolosov’s constant for the implant material.

*Non*-*interacting micro*-*cracks* When the distance between non-contact zones is relatively large compared to *λ*, the interaction parameter $I_{k} \left( {\frac{a}{\lambda }} \right)$ may be taken equal to 1, which leads to the effective contact stiffness $K_{{c , \,{\text{non-int}}}}^{\text{LEK}}$ in the non-interaction case:

18 $$K_{{c ,\,{\text{non-int}}}}^{\text{LEK}} = M_{k} \left( {\alpha , \beta } \right) \times K_{{c ,\,{\text{non-int}}}}^{\text{homog}}$$

#### Kachanov’s model

As shown in Fig. 9c, in the Kachanov’s model, the non-contact zone is modeled by a circumscribed elliptical cavity with $a$ and $b$ being the semi-major and semi-minor axes, respectively. This model neglects interactions between voids. The aforementioned elliptical cavity is embedded in a homogenized medium whose mechanical properties are given by the homogenization of the isotropic mechanical properties of implant and bone materials [58]. The closed-form analytical expression for the effective Young’s modulus $E_{\text{eff}}$ of a two-dimensional isotropic matrix with an embedded elliptical hole randomly oriented is [53]:

19 $$E_{\text{eff}} = \frac{{E_{0} }}{1 + 3p + q},$$

where the microstructural parameters *p* and *q* are the porosity and the average eccentricity over the bone–implant area $A = 2\Delta \lambda$ and are expressed by:

20 $$\begin{aligned} & p = \frac{\pi }{A}ab, \\ & q = \frac{\pi }{A}\left( {a - b} \right)^{2} . \\ \end{aligned}$$

In Eq. (19), *E*_*0*_ corresponds to the equivalent Young’s modulus of the homogenized matrix in which the elliptical hole is embedded and based on plane strain assumption, it is given by:

21 $$E_{0} = \frac{1}{2}\left( {\frac{1}{{E_{i}^{*} }} + \frac{1}{{E_{b}^{*} }}} \right),$$

where $E_{i}^{*}$ and $E_{b}^{*}$ are defined above. The effective contact stiffness of the BII obtained with the Kachanov’s model is given by:

22 $$K_{c}^{\text{KACH}} = \frac{{E_{\text{eff}} }}{2\Delta }.$$

## Data Availability

The datasets used, generated and/or analyzed during the current study are available from the corresponding author on reasonable request.

## Abbreviations

BII: bone–implant interface
FE(M): finite elements (method)
BIC: bone–implant contact ratio
ROI: region of interest
I: interacting micro-cracks (Lekesiz’s scheme)
NI: non-interacting micro-cracks (Lekesiz’s scheme)
